# List of Diseases at the Bath City Dispensary, from September 1, to October 1, 1801

**Published:** 1801-11

**Authors:** 


					388 Diseases in the Bath City Dispensary.
List of Diseases at the Bath City Dispensary, from September r,
to Ottober I, 1801.
Angina ------ i
Aflhma ------ i
Anafarca ------ 3
Althenia - - - - - - 1
Bowel Complaints - - - 5
Contufion - - - - - 2
Cholera ------ 1
Chlorolis ------ 3
Eruptiones ----- 2
Fever (Ample) - - - - 9
Gaftrodynia --/--- 4.
Hernia ------ 3
Hasmorrhagia - - - - 2
Hasmorrhoids - - - - 1
Herpes ------ I
Idleris --- --- - j
Inflammation - - - - 1
Lepra - - - - - 2
Menorrhagia - - - - 3
Nephritic Complaints - - 1
Ophthalmia ----- 1
Pertuflis ------ 3
Phthifis ------ 7
I
Pulmonary Complaints - - 2
Paralyfis ------ 3
Pfora - -- -- -- 1
Proiapfus Ani - - - - 1
Rheumatifm - - 10
Scarlatina ----- t
Synochus Biliofa - - - 36
Pleuritica - - 4
Gaftritica - - 1
Cholerica - - 1
? Dyfenterica - - 3
? - I&erica - - - 1
Anafarca - - 1
Suppuration ----- 2
Syphilis -------
Strain j
Scrofula - - - - r **'. - 2
Stridtures in Urethra - - 1
Typhus - - - - - - 1
Tuffis - - - - - 1
Tumor - - f, - - - 1.
Tinea Capitis ^ - - - - 2
Ulcerated Legs - - - - 7
. ?

				

